# The Research Progress of DNA Methylation in the Development and Function of the Porcine Placenta

**DOI:** 10.3390/ijms251910687

**Published:** 2024-10-04

**Authors:** Zhiyuan Zhang, Jiawei Su, Jiaming Xue, Liyao Xiao, Linjun Hong, Gengyuan Cai, Ting Gu

**Affiliations:** 1 National Engineering Research Center for Breeding Swine Industry, College of Animal Science, South China Agricultural University, Guangzhou 510642, China; zhiyuanzhang@stu.scau.edu.cn (Z.Z.); 20232024019@stu.scau.edu.cn (J.S.); jiamingxue@stu.scau.edu.cn (J.X.); xlyao@stu.scau.edu.cn (L.X.); linjun.hong@scau.edu.cn (L.H.); cgy0415@scau.edu.cn (G.C.); 2 Guangdong Provincial Key Laboratory of Agri-Animal Genomics and Molecular Breeding, College of Animal Science, South China Agricultural University, Guangzhou 510642, China

**Keywords:** pig, DNA methylation, placental development

## Abstract

The pig is the most widely consumed domestic animal in China, providing over half of the meat supply in food markets. For livestock, a key economic trait is the reproductive performance, which is significantly influenced by placental development. The placenta, a temporary fetal organ, is crucial for establishing maternal–fetal communication and supporting fetal growth throughout pregnancy. DNA methylation is an epigenetic modification that can regulate the gene expression by recruiting proteins involved in gene silencing or preventing transcription factor binding. To enhance our understanding of the molecular mechanisms underlying DNA methylation in porcine placental development, this review summarizes the structure and function of the porcine placenta and the role of DNA methylation in placental development.

## 1. Introduction

As an indispensable temporary organ for gas and nutrient exchange between the mother and fetus, the placenta plays a pivotal role in providing nutrition and oxygen, removing metabolic waste, and exhibiting immune and endocrine functions during embryonic development. Consequently, placental development is directly linked to fetal growth and significantly influences the outcome of a pregnancy [1,2,3]. Placental development is a complex physiological process regulated by various factors, including epigenetic factors such as histone modification, DNA methylation, RNA methylation, and non-coding RNAs. DNA methylation, a widespread epigenetic modification found in gene coding and promoter regions, is instrumental in embryonic development, aging, tumorigenesis, and placental development and function [4,5]. This review systematically summarizes the effects of DNA methylation on the structure and function of the porcine placenta, aiming to enhance our understanding of its role, providing a theoretical foundation for future research. Additionally, we provide a comprehensive understanding of how genetic factors, gene expression, and metabolic processes influence the development of the pig placenta, as well as the growth and development of the fetus and piglets. Our goal is to offer valuable insights to researchers and industry professionals, enabling them to refine breeding strategies and production methods, increase fetal survival through improved placental development, foster sows to produce healthier piglets, and ultimately maximize the economic benefits of agricultural production.

## 2. Pig Placenta

### 2.1. The Main Structure of the Pig Placenta

Based on gross morphology, the placenta can be classified into four types across various mammalian species: diffuse (chorionic villi uniformly cover the entire endometrial surface), cotyledonary (chorionic villi coalesce into rounded plaques), zonary (chorionic villi are concentrated around the middle portion of the endometrium), and discoidal (chorionic villi are partially smooth and partially in contact with each other and the endometrium). Additionally, the placenta can be categorized into four types according to the cellular composition of the interhemal region: epithelial chorionic, syndesmochorial , endothelial chorionic, and hemotrichorial. Furthermore, another classification system considers the extent of fetal placental invasion into the endometrium, the degree of endometrial tissue destruction, and the degree of endometrial detachment: deciduate and indeciduate placenta [6,7].

**Figure 1:**
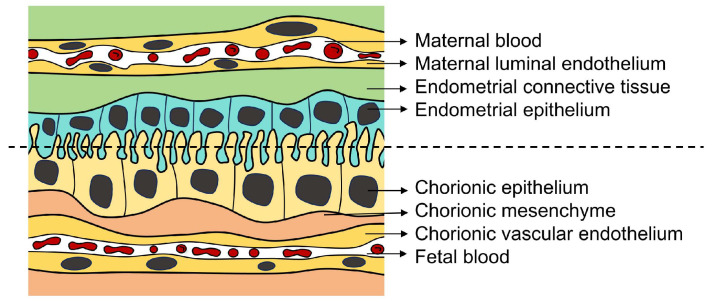
Diagram of the structure of the porcine placental barrier: maternal side above the dividing line and fetal side below the dividing line.

The porcine placenta is classified as a diffuse, epithelial chorionic, and indeciduate placenta [8]. In this type of placenta, chorionic villi are distributed across the surface of the chorionic membrane, with blood vessels situated within the chorionic villi. Notably, there are minor variations in the abundance of chorionic villi among different placental regions [6]. The epithelial chorionic villus placenta is characterized by fetal chorionic trophoblast cells that are in direct contact with the epithelial cells of the maternal endometrium. Importantly, both the chorionic villus and the endometrium remain intact in the placenta, maintaining their independence [9,10]. The term ‘indeciduate placenta’ signifies that the embryo’s allantoic–chorionic membrane does not invade the maternal endometrium, resulting in a fetus that can be delivered without causing a uterine wall hemorrhage [7]. The interhaemal barrier of the porcine placenta comprises the cellular layers separating the fetal and maternal blood circulations. On the maternal side, this barrier consists of the maternal luminal endothelium, endometrial connective tissue, and endometrial epithelium. On the fetal side, it includes the chorionic epithelium (trophoblast), chorionic mesenchyme, and chorionic vascular endothelium [11,12] (Figure 1).

### 2.2. Functions of the Placenta in Embryonic Development

The placenta relies on the expansion of the trophoblast in the uterine cavity to establish pregnancy and maintain embryonic and fetal growth. The rapid development of trophoblast cells promotes the placental surface area and volume, facilitating the transportation of nutrients to the fetus during pregnancy [13,14]. Pigs are multiparous animals, and the development of the placenta is closely related to swine’s reproductive traits, such as the number born alive (NBA) [15]. Placental dysplasia can lead to fetal miscarriage, intrauterine growth retardation, stillbirth, and heart defects, among other developmental anomalies [16,17,18,19,20,21].

The growth and development of the placenta in utero is a dynamic and mutually coordinated process. During embryonic development, the placenta serves as a transient organ for the exchange of material and information between the mother and the embryo, mediating the transfer of nutrients such as proteins, lipids, carbohydrates, vitamins, minerals, and other nutrients, as well as oxygen and immune molecules, from the mother to the fetus [1]. The placenta also secretes metabolic hormones such as placental prolactin and placental growth hormone, which promote maternal glucose and lipid metabolism and, ultimately, fetal growth and development [22].

**Figure 2:**
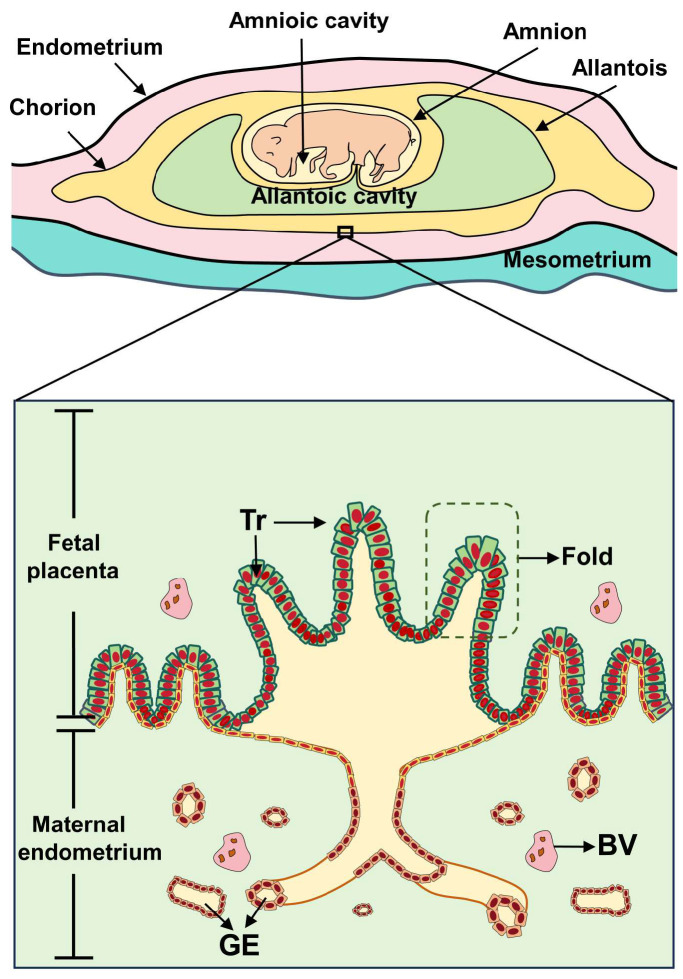
Diagram of the morphologic structure of the pig placenta. Tr: trophoblast; GE: Glandular epithelial cells; BV: blood vessel.

During placental development, the placental trophoblastic epithelium and the uterine endometrial epithelium adhere to each other, and these two membranes fold to form the placental folds and the chorionic villi in the folds, which are the basic functional units for the exchange of material between the fetus and the mother [3]. Dysfunction of the trophoblast cells of the placenta, insufficient vascularity with abnormal fold morphology, and inefficient transit are all potential factors contributing to fetal developmental disorders [23] (Figure 2).

## 3. DNA Methylation

DNA methylation is one of the most prevalent epigenetic modifications, including histone modification, RNA methylation modification, and non-coding RNA regulation. Epigenetic modifications have been shown to play significant roles in regulating biological processes without altering the underlying genomic DNA sequence [24,25,26].

### 3.1. Concept of DNA Methylation

**Figure 3:**
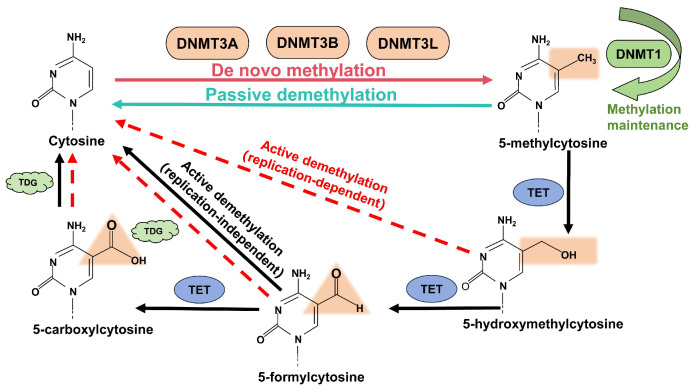
Diagram of DNA methylation and demethylation mechanisms.

DNA methylation modification involves the addition of methyl groups (-CH_3_) to the cytosine bases of DNA, primarily occurring at the CpG site [27,28]. DNA methylation changes can be categorized into three phases: writing, maintenance, and erasure (Figure 3). Methyl-transferases, such as DNA (cytosine-5) methyltransferase 3 alpha (DNMT3A), DNA (cytosine-5) methyltransferase 3 beta (DNMT3B), and DNA (cytosine-5) methyltransferase 3-like (DNMT3L), are primarily responsible for the writing of DNA methylation, with DNMT3A and DNMT3B directly catalyzing the methylation of unmethylated DNA strands [29]. DNMT3L, although lacking catalytic activity, can bind to DNMT3A and DNMT3B to enhance their functions [30,31]. The maintenance of DNA methylation is primarily mediated by the methyl-maintaining enzyme DNMT1, which preferentially targets hemimethylated DNA for conversion to methylated DNA [32]. The Ten-eleven translocation (TET) family is primarily responsible for DNA methylation erasure. Three members of the TET protein family (TET1, TET2, TET3) can convert 5-methylcytosine (5mC) to 5-hydroxymethylcytosine (5hmC), a crucial modification for genomic reprogramming in mammalian primordial germ cells and early embryonic development [33,34,35]. During base excision repair, Thymine DNA Glycosylase (TDG), a member of the uracil DNA glycosylase family, can perform G/T mismatch repair, which can repair 5mC:G to unmodified C:G [36].

### 3.2. Detection Techniques of DNA Methylation

DNA methylation research can be conducted at both the whole-genome and specific methylation site levels. Techniques such as methylated DNA immunoprecipitation sequencing (MeDIP-seq), whole-genome bisulfite sequencing (WGBS), next-generation sequencing (NGS), and nanopore sequencing can be utilized to detect methylation throughout the genome. However, NGS alone cannot directly identify 5mC. It typically relies on methods like methylation-specific PCR (MS-PCR) or bisulfite sequencing PCR (BSP), which convert unmethylated cytosines into uracil. This conversion enables the differentiation between methylated and unmethylated sites during sequencing [37,38,39,40,41,42].

In addition to BSP and MS-PCR, other methods that are commonly employed for the study of methylation-specific sites include combined bisulfite restriction analysis (COBRA), methylation-sensitive high-resolution amplification (MS-HRM), methylation-sensitive single nucleotide primer amplification (MS-SnuPE), reduced representation bisulfite sequencing (RRBS), and oxidative-reduced representation bisulfite sequencing (oxRRBS) [43,44,45,46,47,48,49,50,51,52,53,54,55,56,57].

In addition to methylation-specific sites, newly identified methylation sites, referred to as neo-methylation sites, can be detected using methyl-CpG binding domain (MBD) column chromatography and DNA microarray techniques. These neo-methylation sites may be involved in yet-to-be-understood mechanisms of gene expression regulation or disease-related methylation changes [58,59,60,61]. Recently, CRISPR-Cas-assisted sensor system technology has emerged as a promising approach for detecting DNA methylation-specific sites [62,63].

The ability to differentiate between 5mC and 5hmC varies among different DNA methylation detection methods. While 5mC is the predominant form of DNA methylation and is typically associated with gene silencing, 5hmC is an active metabolic derivative of 5mC that may be involved in regulating gene expression. The presence of and fluctuations in 5hmC levels can reflect cellular developmental and differentiation states, as well as being closely linked to disease progression. Therefore, accurate detection and differentiation between 5mC and 5hmC are essential for understanding gene regulation and the underlying mechanisms of diseases [36]. During WGBS processing, 5mC remains unchanged, while 5hmC may be erroneously identified as unmethylated, hindering the ability to distinguish between the two. RRBS employs restriction endonucleases to fragment DNA and enrich for CpG islands prior to sequencing. However, as it relies on bisulfite treatment, RRBS cannot effectively differentiate between 5mC and 5hmC. In contrast, oxRRBS first oxidizes 5hmC to 5-formylcytosine (5fC), enabling these oxidized bases to be effectively differentiated during bisulfite treatment, thereby allowing for the distinction between 5mC and 5hmC. Other specific methods are often designed for targeted loci and may lack whole-genome coverage, rendering them incapable of differentiating between these two modifications as well [57,64]. Therefore, WGBS and RRBS have significant limitations in distinguishing between 5mC and 5hmC, while oxRRBS offers a more effective means of differentiation through chemical processing.

### 3.3. Regulatory Mechanisms of DNA Methylation

DNA methylation is a fundamental mechanism regulating gene expression, primarily occurring within repetitive DNA elements, centromeres, telomeres, and promoters and enhancers. It plays a critical role in preserving genome integrity. Nevertheless, the contribution of methylation within promoters and enhancers to the overall 5mC content is relatively modest [65,66].

Currently, DNA methylation primarily regulates the gene expression through the following mechanisms: (1) It alters DNA molecular conformation. DNA methylation can not only influence the interaction between gene transcription factors and the upstream regulatory region of the gene but also indirectly mediate transcriptional inhibition by modifying the chromatin structure [26,67]. (2) It carries out spatial positional blocking. The methyl group of 5-methylcytosine can hinder the transcription factor binding to DNA, thereby interfering with the normal function of transcription factors [26]. (3) It influences protein interaction patterns. DNA methylation can also recruit methylation-sensitive proteins, such as MeCP2, which further bind to other chromatin-modifying proteins to create an inhibitory chromatin environment, further suppressing the gene expression [68,69]. (4) It inhibits gene transcription. By increasing the stability of the DNA double-helix structure, which impedes the unwinding and unstranding of the DNA, it alters the gene regulatory region structure, maintaining chromatin in a condensed state, which is unfavorable for transcription [70].

In addition, DNA methylation interacts with other epigenetic marks, such as histone modifications, to fine-tune the gene expression, ensuring that cells maintain their daily functions and biological activities according to tissue-specific physiological needs [71,72].

## 4. Effects of DNA Methylation on Placental Structure and Function

DNA methylation maintains precise and orchestrated gene expression and is essential for mammalian embryo implantation and placental development [73,74,75,76,77,78,79,80]. In pigs, DNA methylation patterns are established during early embryogenesis, while most methyl groups are erased during post-fertilization to blastocysts. They then undergo de novo methylation from about the 8- to 16-cell stage [78]. DNA methylation can affect the development of the porcine placenta by affecting the vascular formation and fold generation, while energy metabolism and immune metabolism processes are regulated by DNA methylation on the encoding genes for regulatory factors [79,81,82,83,84,85,86,87,88].

### 4.1. DNA Methylation Affects Placental Vascular Development

Porcine placental vascular development occurs during the early stages of gestation and involves vasculogenesis and angiogenesis. Vasculogenesis is the formation of new blood vessels, including the differentiation of pluripotent mesenchymal cells into hematopoietic stem cells to create the initial vascular network. Angiogenesis is the process of forming new blood vessels from existing capillaries through the migration and proliferation of endothelial cells in both branching (via germination to form new blood vessels) and non-branching (via elongation to form capillary rings) patterns [89,90].

Neuropilin 2 (*NRP2*), a member of the neuropilin receptor protein family, functions as a coreceptor for vascular endothelial growth factor (VEGF) in vascular development. Concurrently, Sonic hedgehog (*SHH*) can regulate endothelial cell growth, promote cell migration, and stimulate neointima formation [91,92,93]. In intrauterine growth retardation (IUGR) placentas, compared to normal birth weight (Normal) placentas, the promoter regions of the NRP2 and SHH genes were hypermethylated, and their expression levels were negatively correlated with methylation levels. These findings indicate that NRP2 and SHH are expressed at lower levels in IUGR compared to normal conditions, suggesting that a decreased expression of NRP2 and SHH is detrimental to the process of placental vasculogenesis [81].

VEGF promotes endothelial cell proliferation and migration by binding to its receptors (VEGF-R1 and VEGF-R2) and can regulate placental trophoblast cell proliferation, vascular structure formation, angiogenesis, and vascular permeability [6,94,95,96]. The placental and umbilical cord blood vessels serve as the primary conduits for nutrient exchange and waste product transport between the embryo and the mother. Decreased VEGFA expression can lead to abnormal placental development [97]. A comparative analysis of placentas from fetuses with the highest birth weight (HBW) and lowest birth weight (LBW) within the same litter revealed distinct morphological characteristics of porcine placental tissues, as observed through hematoxylin–eosin (H-E) staining. HBW placentas exhibited a higher concentration of nucleated red blood cells within the vessels and intervillous spaces of the chorionic villi compared to LBW placentas. Moreover, LBW placentas demonstrated higher methylation levels of VEGFA and VEGF-R2 than HBW placentas, with an inverse correlation between the expression levels and methylation levels. These findings suggest that angiogenesis and vascular permeability are reduced in LBW placentas [82,98]. DNA methylation-mediated silencing of VEGF-R1 prevented homodimer and heterodimer binding to the VEGF receptor family. Song et al. observed hypermethylation of VEGF-R1 in the placenta of aborted piglets, accompanied by lower expression levels than healthy placentas [84]. Han Yang also found that VEGF-R1, when expressed at significantly low levels in the placenta (*p* < 0.05) of porcine parthenogenetic (PA) female fetuses, can impair the angiogenesis process and result in defective placental development [99].

Rap1 is a small GTPase, and Rap1 signaling promotes vascular development [100]. *VAV1*, *ITGB3*, and *ITGB1* are genes within the Rap1 signaling pathway and exhibit lower expression levels in the placenta of piglets with LBW within the same litter. Additionally, these genes are highly methylated. Experimental validation using 5-Aza-2′-deoxycytidine-induced demethylation of PTr2 cells suggests that DNA methylation can hinder placental angiogenesis and vasculogenesis by reducing the expression of *VAV1*, *ITGB3*, and *ITGB1* [82,100]. Placental angiogenesis and blood flow are regulated by various factors within the vasodilator and constrictor systems, such as endothelins (EDNs) and the renin-angiotensin system. Endothelin-2 (*EDN2*) is one of the EDNs, known for its potent vasoconstrictor activity [101]. *EDN2* exhibited lower methylation and higher expression in the placenta at gestation day 35 compared to gestation days 21 and 28, suggesting that EDN2 may be regulated by DNA methylation during placental vascular development and promotes its development [79]. Adrenomedullin (*ADM*), a vasodilator that primarily reduces peripheral arterial resistance, was found to be reduced in IUGR placentas compared to normal placentas, indicating that a low *ADM* expression may contribute to fetal growth retardation. Moreover, *ADM* demonstrated higher levels of methylation modification in the promoter region of the IUGR group, suggesting that DNA methylation-mediated regulation of *ADM* expression leads to increased resistance to blood flow, affecting blood supply and, ultimately, the efficiency of placental material transport [81,102].

### 4.2. DNA Methylation Affects the Development of Placental Folds

The morphology and function of the placenta are critical for normal embryonic development. Increased placental folding not only enhances capillary density and permeability but also expands the area for material exchange between the fetus and the mother, thereby improving the efficiency of placental nutrient transfer [103,104,105]. Studies have revealed that trophoblast cells begin to adhere to the uterine cavity epithelium at 21 days of gestation in pigs, forming a trophoblast–endometrial epithelial double-layered structure. Placental fold formation commences shortly thereafter, with a small number of irregular microscopic folds appearing on the 28th day of gestation. Several studies have demonstrated that placental folds begin to develop after 30 days of gestation, becoming deeper, wider, and more complex by the 35th day compared to the 28th day. By 65 days of gestation, the placental fold morphology tends to be fully developed, with a significant increase in fold width and the number of trophoblast cells compared to 40 days of gestation [79,83,106,107]. The migration and proliferation of trophoblast cells directly determine placental fold development. Angiopoietin-like 4 (ANGPTL4) promotes the vascularization and induces proliferation, migration, and adhesion of porcine trophoblast cells, fostering placental fold development [108]. Placental fold development was more complete in normal placentas than IUGR placentas, suggesting that DNA methylation can benefit placental fold development by promoting ANGPTL4 expression [81]. Rap1 regulates trophoblast cell proliferation, adhesion, and fusion [109,110,111]. Studies have shown that in porcine placentas at 21, 28, and 35 days of gestation, Rap1 exhibits the lowest methylation level and the highest expression level at 35 days, when trophoblast cells and folds are more fully developed. This suggests that a DNA methylation loss at Rap1 may promote placental fold development [79].

Extracellular matrix (ECM)-degrading enzymes can facilitate trophoblast cell proliferation and migration by degrading the ECM, thereby promoting placental fold development [112,113]. Heparinase (*HPSE*) is the only endogenous endoglycosidase that is capable of degrading heparan sulfate proteoglycans (a major ECM component), and it plays a role in ECM remodeling, cell migration, and growth [114]. Morphological studies of porcine placentas at 26, 50, and 95 days of gestation have suggested that *HPSE* may contribute to the development of porcine placental folds [115]. Subsequent research has revealed that *HPSE* is highly expressed after 26 days of gestation but becomes nearly undetectable after 50 days of gestation [116]. In alignment with this finding, another study observed upregulated *HPSE* expression at 21 and 28 days of gestation, followed by near-undetectable levels at 35. This expression pattern exhibited a strong negative correlation with DNA methylation (R = −0.99984), suggesting that *HPSE* gene transcription might be regulated by DNA methylation. Collectively, these studies demonstrate that DNA methylation can promote the attachment, migration, and folding of endometrial epithelial bilayer structures by regulating HPSE expression, thereby facilitating the formation of porcine placental folds [79,116].

### 4.3. DNA Methylation Affects Placental Metabolic Function

The placenta is a vital organ for embryonic growth and survival. Abnormal placental metabolic function can lead to decreased material transport between the mother and the fetus, resulting in serious adverse consequences for the fetus [6,14,117,118]. Fatty acids (FAs) serve as a primary energy source for placental development and are essential components of the placental structure. Maternal regulation of fatty acid metabolism is crucial for the proliferation of placental trophoblast cells and vascular development [119,120,121].

Fatty acid transporter proteins (FATPs), fatty acid translocase (FAT/CD36), and cytoplasmic fatty acid binding proteins (FABPs) are the primary fatty acid carriers involved in regulating fatty acid uptake and transport in the placenta [122,123,124,125]. Yang et al. intervened in placental trophoblast cells using a nitric oxide synthase inhibitor (L-NAME), observing a significant increase in lipid content and a marked upregulation of intracellular *FABP4* expression (*p* < 0.05). Concurrently, the methylation level of the *FABP4* gene promoter region and the expression level of DNMT1 were significantly reduced, suggesting that DNA methylation may participate in L-NAME-induced regulation of placental lipid metabolism by modulating *FABP4* expression [126]. In a study examining pig placentas at gestational days 40, 65, and 95, the expression levels of *CD36*, *FATP4*, and *FABP5* were significantly upregulated by 2.8-fold, 5.6-fold, and 12.0-fold, respectively, between gestational days 40 and 95. Insulin-like growth factor 2 (IGF2) promotes the metabolic activities of various cell types. The transcript levels of IGF2 were significantly upregulated, and DNA methylation was reduced in the placenta at 95 days of gestation compared to 65 days of gestation. Furthermore, the overexpression of IGF2 significantly upregulated the expression of *CD36*, *FATP4*, and *FABP5* in PTr2 cells, suggesting that these proteins may be key regulators of long-chain fatty acid (LCFA) transport in the porcine placenta. Additionally, IGF2 might be regulated by DNA methylation, influencing the expression of fatty acid carriers and thereby participating in placental fatty acid metabolism [83,127].

Protein kinase cGMP-dependent 2 (*PRKG2*) is a cGMP-dependent protein kinase involved in cellular signaling and the regulation of various biological processes, playing a crucial role in energy metabolism and cell proliferation [128]. Phosphoenolpyruvate carboxykinase 1 (*PCK1*) is a key enzyme in gluconeogenesis, responsible for converting oxaloacetate to phosphoenolpyruvate and contributing to energy metabolism. *PCK1* helps maintain blood glucose levels and energy supply [129]. In pig placentas from larger litter size groups (LLGs) and smaller litter size groups (SLGs), the expression levels of *PRKG2* and *PCK1* were higher in LLGs, suggesting that the well-developed piglets may be attributed to the lower methylation and higher expression of *PRKG2* and *PCK1* in the placenta, which enhanced the energy metabolism function of the placenta [88,130,131,132].

### 4.4. DNA Methylation Affects Placental Immune Function

The inflammatory process caused by the infiltration of immune cells, as well as the signaling proteins produced and secreted by immune cells, can regulate the implantation of the embryo and the development of the placenta [133]. Abnormal DNA methylation can affect the proliferation, differentiation, and apoptosis of immune cells, thereby affecting the synthesis and secretion of inflammatory factors, which in turn affects the immunoregulatory function of the placenta and is involved in mediating the onset and progression of a variety of immune diseases [134].

Interleukin-1 (*IL-1*) activates and regulates T and B lymphocyte activation, proliferation, and differentiation and plays a vital role in the inflammatory response [135,136]. There are two main isoforms of *IL-1α* and *IL-1β*, as well as two receptors, namely interleukin-1 receptor type 1 (*IL1R1*) and interleukin-1 receptor type 2 (*IL1R2*) [137,138]. It was found that *IL1R2* was less expressed and more methylated in HBW placentas than in LBW placentas, suggesting that the immune activity in the placenta was affected by the reducing *IL1R2* expression that favors placental development [82].

Annexin A1 (*ANXA1*) is a protein expressed on cell membranes that are involved in membrane binding and cell signaling, which can transmit inflammatory signals to inflamed tissues and attenuate the inflammatory response by binding to cortisol [139]. It was found that the expression of *ANXA1* was significantly downregulated in IUGR compared to Normal. The deficiency of *ANXA1* resulted in a lack of anti-inflammatory response in the IUGR placenta when *ANXA1* was methylated at a higher level in IUGR, suggesting that the expression of *ANXA1*, probably repressed by DNA methylation, inhibited fetal development [81].

Polymorphic retrotransposon element 1 (*PRE-1*) is a short interspaced nucleotide element short interspersed nuclear elements (*SINEs*); PRE-1 and filamentous satellite DNA exist as highly repetitive sequences in the heterochromatin regions of pigs, which are capable of indirectly affecting immune function by influencing gene expression, genome stability, and immune system regulation, and the methylation levels of these two sequences can reflect the genome-wide methylation status of pigs [140,141]. Studies found that the methylation modification levels of *PRE-1 SINE* and satellite sequences were abnormally increased in aborted porcine placentas. Thus, the abnormal methylation levels of placentas might lead to fetal developmental defects and abortion [85,86].

### 4.5. DNA Methylation Affects Regulatory Factors in the Placenta

DNA methylation can also influence placental development by regulating regulatory factors. Pleckstrin homology-like domain, family A, member 2 (*PHLDA2*) is a key regulatory factor with functions such as regulating cell signaling, intracellular transport, and others [142]. Placentas from embryos with functional deficiencies in the *PHLDA2* gene exhibit overdevelopment and increased glycogen storage, whereas placentas from embryos with *PHLDA2* gene overexpression are retarded compared to wild-type placentas [143,144]. Researchers investigated the *PHLDA2* expression in the placentas of goats at 30, 45, 60, and 90 days of gestation, finding that *PHLDA2* exhibited the highest gene expression and the lowest level of methylation in the promoter region at 45 days of gestation. This suggests that *PHLDA2* expression is influenced by DNA methylation during gestation [145].

In human placentas, the expression of *PHLDA2*, along with the epigenetic regulators DNMT1, DNMT3A, DNMT3B, and TET3, was significantly elevated in cases of IUGR compared to non-IUGR placentas, confirming that excess *PHLDA2* adversely affects normal placental development. Although no differences in DNA methylation levels were observed between the IUGR and control groups, dysregulation of epigenetic mechanisms and increased expression of imprinted genes and epigenetic regulators similarly impact fetal development and result in the IUGR condition [146]. As a pivotal epigenetic regulator, DNMT3B is essential for maintaining the repression of germline genes in the trophoblast lineage of mouse placentas. Studies have demonstrated that using Sox2-Cre to remove DNMT3B in embryos enables the complete expression of genes in trophoblast cells, allowing embryos to survive to birth. Consequently, de novo DNA methylation by DNMT3B is crucial for placental development and embryo survival [147].

Placenta growth factor (*PLGF*) is a key regulator secreted by the placenta, promoting vascular endothelial cell proliferation, placental angiogenesis, resistance to vascular endothelial cell apoptosis, trophoblast proliferation and invasion, and increased vascular permeability [82,148,149,150,151]. Importantly, qPCR experiments revealed significantly lower PLGF expression in porcine PA placentas compared to the control group (*p* < 0.05), suggesting that an early developmental blockage of PA embryos might be attributed to low PLGF expression [99]. In pre-eclampsia, the methylation status of PLGF demonstrated decreased methylation levels compared to normal controls, indicating that the level of DNA methylation modification of angiogenic factors in the placenta is regulated in pre-eclampsia and influences the pregnancy outcome. The DNA methylation level of *PLGF* in the placenta of human patients with early-onset pre-eclampsia (EOPE) was found to be significantly lower than that of late-onset pre-eclampsia (LOPE) cases and uncomplicated controls. This suggests that the PLGF expression is influenced by DNA methylation, implying that DNA methylation can modulate the pathophysiological processes of EOPE by regulating the *PLGF* expression [152].

In summary, DNA methylation plays a multifaceted role in regulating porcine placental development and function, ultimately influencing both embryo survival and fetal health status after delivery, as summarized in Table 1.

## 5. Summary and Outlook

In conclusion, DNA methylation can regulate porcine placental development by influencing trophoblast cell growth, placental vascular and fold formation, energy metabolism processes, and immune metabolism processes. Aberrant DNA methylation can result in embryo attachment failure, fetal developmental defects, and miscarriage. Further mechanistic investigations are required to elucidate the roles of DNA methylation in regulating porcine placental development. Additionally, the interplay between DNA methylation and other epigenetic modifications remains unclear, potentially offering novel strategies for optimizing breeding strategies and improving animal husbandry production management.

## Figures and Tables

**Table 1:** Summary of the effects of DNA methylation in regulating the development of the porcine placenta.

Breed	Sample Source	Method	Genes	Methylation State	Possible Target	Reference	Year
Rongchang pig	IUGR and Normal	DNA methylation and transcriptome	ANXA1	Hypomethylation in Normal	Inflammatory response	[81]	2024
			ANXA1		Development of trophoblast cells		
			ADM				
			NRP2, SHH, and ADM		Development of vascularity		
Duroc sow	HBW and LBW		VAV1, ITGB3, and ITGB1	Hypomethylation in HBW	Development of vascularity	[82]	2024
Tibetan pig	Days 21, 28, and 35 of pregnancy		HPSE	Hypomethylation on days 21 and 28	Development of placental folds	[79]	2023
			Rap1	Hypomethylation on day 35	Development of trophoblast cells		
			EDN2, APLN, and ALDH2		Development of vascularity		
Duroc	Days 40, 65, and 95 of pregnancy	DNA methylation	IGF2, CD36, FATP, and FABP5	Hypomethylation on day 95	Fatty acid metabolism	[83]	2023
Berkshire pig	SLG and LLG	DNA methylation and transcriptome	PRKG2	Hypomethylation in LLG	Energy metabolism	[88]	2017
			CLCA4		Development of vascularity		
			PCK1		Energy metabolism		
Unknown	PA and normal	DNA methylation	VEGF-R1	Hypermethylation in PA	Development of vascularity	[84]	2017
Yorkshire	Artificial insemination and aborted cloning		PRE-1	Hypermethylation in aborted clones	Development of defects and abortions	[85]	2014
			H19	Hypomethylation in aborted clones			
Unknown	PA and normal		H19	Hypomethylation in PA	Implantation failure and abortion	[86]	2013
Northeast-min pig	Dead clones and live cloning		IGF2, PEG3, and GRB10	Hypermethylation in dead clones	Development of trophoblast cells	[87]	2010

## Data Availability

The original contributions presented in this review are included in the article within the text; further inquiries can be directed to the corresponding author.
